# Retracted: The Role of Tomographic Ultrasonography in Conduit Mapping before Coronary Artery Bypass Grafting

**DOI:** 10.1155/2020/7825329

**Published:** 2020-11-24

**Authors:** Radiology Research and Practice

*Radiology Research and Practice* has retracted the article titled “The Role of Tomographic Ultrasonography in Conduit Mapping before Coronary Artery Bypass Grafting” [1]. An investigation completed by the University of Manchester concluded that the author of the article did not possess the appropriate permissions for the publication of the data. It is therefore being retracted with the agreement of the journal and the University of Manchester. The author, Syed Mohammad Asim Hussain, does not agree to the retraction.
